# Learning from challenges to maternal health during COVID Era; Perspective from Pakistan

**DOI:** 10.1016/j.amsu.2022.104682

**Published:** 2022-09-15

**Authors:** Faizan Fazal, Mohammad Ebad Ur Rehman, Sumia Fatima, Shagufta Saeed Sial, Tayyaba saleem, Jawad Basit, Tehseen haider, Sajeel Saeed, Haris Mustafa

**Affiliations:** Department of Medicine, Rawalpindi Medical University, Block E Satellite Town, Rawalpindi, Pakistan

**Keywords:** Maternal health, COVID

The SARS-COV2 variant of the Corona Virus caused a great economic and societal impact and sparked an unheard-of pandemic in the year 2020. COVID-19 was declared as a public health emergency on 30th January, 2020 [1]. It destabilized the economies of several countries and altered the state of global health. There have been 579 million confirmed cases and 6.4 million deaths globally as of August 5, 2022 [2]. Worldwide health crisis and devastation due to COVID-19 served as a forewarning to the world about how vulnerable humans are to infection all over the world [3]. Prevention remained one of the main strategies to curb COVID-19 spread. In a review on evidence based management for COVID-19 by Riaz agha et al. it was shown that transmission of COVID-19 can be reduced or prevented by Hand hygiene, covering nose and mouth during coughing and sneezing, using high-filtration masks including N95, goggles, and gowns [4].

Pregnant women and their fetuses constitute a high-risk population when infectious disease outbreaks, such as the current COVID-19 Pandemic [6]. Maternal and neonatal health are affected both directly through infection itself but also indirectly due to changes in health care accessibility and socioeconomic disruptions [5]. The mothers are found to have a higher probability of developing more severe symptoms [6]. Women with COVID-19 diagnosis had higher rates of pregnancy-induced hypertension (RR, 1.46), preeclampsia/eclampsia (RR, 1.76), and infections requiring antibiotics (RR, 3.38). Moreover, there is an association with a greater risk of admission to ICU/high-dependency unit (RR, 5.04) and referral to a higher level of care (RR, 6.07). Among all ICU admissions, women with COVID-19 diagnosis stayed 3.73 days longer than women without COVID-19 diagnosis [7]. With COVID 19, fetal brain faced neurodevelopmental challenges in a cytotoxic environment of inflammation and increased catecholamine levels, while the mothers were faced with a hostile environment of uncertainty surrounding the viral spread, rich in bio-social threats that were detrimental to both maternal and fetal health, as well as to Public Health [8].

Regarding the indirect impact of Covid-19 due to social and economic circumstances, significant increase in maternal mental health issues, such as clinically relevant anxiety and depression have been reported [3]. Domestic violence(DV) seemed to be on the rise. There was an increase of 200 percent in DV cases in Pakistan amidst COVID-19 [9]. Reduced prenatal care visits contributed to higher rates of mortality and lasting impairments, while working moms struggled to meet rising childcare demands [6]. During lockdown, the number of children treated for pneumonia, diarrhea and malaria decreased due to decreased visits to health care settings [5,7]. Children missed vaccine doses as a result of decreased vaccination coverage and a decrease in the overall number of immunizations administered [10].

All of these changes caused an increase in maternal health problems and adverse fetal and newborn outcomes, likely due to delayed care-seeking behavior [3]. The first wave (Jan to May 2020) witnessed a greater decline in treatment utilization in Pakistan, with children under 5 treated for pneumonia experiencing the greatest decrease (82%). The number of hospital deliveries and first postnatal visits decreased (37% each) followed by the number of caesarean sections (57%). From June through September, service use improved, but the COVID-19 second wave (October to December 2020) caused another decline [3].

There was a global shift to telemedicine to minimize actual physical or face-to-face contact [9]. Anesthetists for instance evaluated patients who were electively scheduled for caesarean section by video calls, and obstetricians monitored patients’ blood pressure and sugar profiles remotely from home [11]. Prenatal stress and anxiety were less commonly observed in mothers who had received tele-education from psychologists throughout the pandemic [11]. However, a number of critical issues, including illiteracy, poverty, limited internet access, and ethnic minority must be taken into account as potential obstacles for telecommunication. To meet the demand, coordinated efforts must be made to update all connected technology and expand internet access [9].

Moreover, the health system should direct its approach to community-based service to reach neglected women and children who had not arrived at the facility and who cannot afford telemedicine due to poverty, and limited internet access [11]. It should also mobilize its workforce and resources to the lowest service delivery points and health posts [12]. This is especially challenging for a resource depleted country like Pakistan but with careful planning we can administer health services remotely to women from low income households living in the rural communities.

During the COVID-19 pandemic the economic issues also prevailed. There was a lack of both supplies and medications to support maternal and child health. The healthcare system needs to be reassessed and improved in order to prevent recurring problems [13]. In a review on socioeconomic implications of COVID-19 pandemic, it was said that Covid-19 has caused the need for medical supplies to be increased [14]. This is especially challenging for a resource depleted country like Pakistan but with careful planning we can administer health services remotely to women from low income households with limited access to healthcare.

Though the number of COVID cases have declined but it is expected to see a spike in COVID-19 cases. This is due to Omicron sub-variants like BA.4 and BA.5 this summer, as the coronavirus is quickly mutating [15]. We should not let a crisis go to waste as the globe re-strategizes for the post-2020 period. This crisis provides us an opportunity to set rules and allocate resources for future occurrences [10].

## Please state any sources of funding for your research

None

## Ethical Approval

Not applicable

## Consent

Not applicable

## Author contribution

Faizan Fazal : Study conception, write-up, critical review and approval of the final version, Mohammad Ebad Ur Rehman: Study conception, write-up, critical review and approval of the final version,

Sumia Fatima : Study conception, write-up, critical review and approval of the final version, Shagufta saeed sial : Study conception, write-up, critical review and approval of the final version,

Tayyaba saleem : Study conception, write-up, critical review and approval of the final version,

Jawad Basit : Study conception, write-up, critical review and approval of the final version,

Tehseen Haider: Study conception, critical review and approval of the final version,

Sajeel saeed: Study conception, critical review and approval of the final version

Haris Mustafa : Study conception, write-up, critical review and approval of the final version,

## Registration of Research Studies

Name of the registry: Not applicable

Unique Identifying number or registration ID: Not applicable

Hyperlink to your specific registration (must be publicly accessible and will be checked): Not applicable

## Guarantor

Faizan Fazal

Mohammad Ebad Ur Rehman

## Declaration of competing interest

The authors have no conflicts of interest
